# Wearable Robots: An Original Mechatronic Design of a Hand Exoskeleton for Assistive and Rehabilitative Purposes

**DOI:** 10.3389/fnbot.2021.750385

**Published:** 2021-10-21

**Authors:** Nicola Secciani, Chiara Brogi, Marco Pagliai, Francesco Buonamici, Filippo Gerli, Federica Vannetti, Massimo Bianchini, Yary Volpe, Alessandro Ridolfi

**Affiliations:** ^1^Department of Industrial Engineering, University of Florence, Firenze, Italy; ^2^IRCCS Don Gnocchi, Don Carlo Gnocchi Foundation, Firenze, Italy; ^3^Institute for Complex Systems, National Research Council, Sesto Fiorentino, Italy

**Keywords:** wearable robot, hand exoskeleton, telerehabilitation, home assistance, mechatronics design, robotics

## Abstract

Robotic devices are being employed in more and more sectors to enhance, streamline, and augment the outcomes of a wide variety of human activities. Wearable robots arise indeed as of-vital-importance tools for telerehabilitation or home assistance targeting people affected by motor disabilities. In particular, the field of “Robotics for Medicine and Healthcare” is attracting growing interest. The development of such devices is a primarily addressed topic since the increasing number of people in need of rehabilitation or assistive therapies (due to population aging) growingly weighs on the healthcare systems of the nation. Besides, the necessity to move to clinics represents an additional logistic burden for patients and their families. Among the various body parts, the hand is specially investigated since it most ensures the independence of an individual, and thus, the restoration of its dexterity is considered a high priority. In this study, the authors present the development of a fully wearable, portable, and tailor-made hand exoskeleton designed for both home assistance and telerehabilitation. Its purpose is either to assist patients during activities of daily living by running a real-time intention detection algorithm or to be used for remotely supervised or unsupervised rehabilitation sessions by performing exercises preset by therapists. Throughout the mechatronic design process, special attention has been paid to the complete wearability and comfort of the system to produce a user-friendly device capable of assisting people in their daily life or enabling recorded home rehabilitation sessions allowing the therapist to monitor the state evolution of the patient. Such a hand exoskeleton system has been designed, manufactured, and preliminarily tested on a subject affected by spinal muscular atrophy, and some results are reported at the end of the article.

## 1. Introduction

The demographic, economic, social, technological, environmental, and political factors (DESTEP factors) of the last decades of the twentieth century and the first years of the twenty-first century have paved the way to the advent of Robotics for Medicine and Healthcare (Butter et al., 2008). These factors have driven a breakneck growth of robotic systems for medical purposes—equipment, treatment, and rehabilitation. The most significant innovation and development areas are prevention and diagnostics, professional care support, surgery, assistance, and rehabilitation for disabled or chronically ill patients. WHO estimates that over 1 billion people live with disabilities^1^. Such a number is bound to rise because of population aging and the significant increase in chronic disorders (non-communicable diseases), for which, according to WHO^2^, almost 15 million people die every year, and many others lose their mobility functions. Upper-limb functions loss is one of the most impairing disabilities caused by diseases or traumas, and their recovery is seen as a rehabilitation priority (Anderson, 2004; Huang et al., 2017). For this reason, upper-limb devices, hand ones particularly, have exceptional attention in the field of Robotics for Medicine and Healthcare (Duruoz, 2016), which is arising as a powerful tool to overcome some primary limits of the standard Assistive Technology (AT).

Over the years, many different devices have been developed to recover hand functions and restore the life quality of impaired people. Some of these are already commercially available, e.g., *HandyRehab* from Zunosaki^3^, *exomotion*© from HKK Bionics^4^, *Carbonhand* from Bioservo Technologies^5^, or *Neomano* from neofect^6^. Despite their significant variability, such devices have in common some requirements (Sarac et al., 2019), such as: (i) being correctly coupled with the assisted hand; (ii) ensuring user safety and comfort; (iii) being effective in force transmission; and (iv) being as affordable and available as possible. A wide range of Hand Exoskeleton Systems (HESs), achieving at least one of these requirements, are suggested in the literature and can be distinguished according to different aspects, typically the aim, the assisted movements, mechanical design, actuation, and control systems (Troncossi et al., 2016; Meng et al., 2017; Sarac et al., 2019; Desplenter et al., 2020).

Concerning the aim, such devices can have mainly rehabilitative (Dovat et al., 2008; Tong et al., 2010; Ho et al., 2011; Lambercy et al., 2013; Cempini et al., 2014; Polygerinos et al., 2015; Diez et al., 2018; Putzu et al., 2018; Wang et al., 2018; Bouteraa et al., 2019) and assistive (In et al., 2015; Randazzo et al., 2017; Yun et al., 2017; Cappello et al., 2018; Hadi et al., 2018; Yu et al., 2019; Dittli et al., 2020; Yurkewich et al., 2020) purposes. Exoskeletons or end-effector rehabilitation robots are used for treatments—typically in clinical settings—to recover from the loss of motor functions (Maciejasz et al., 2014; Zhang et al., 2018). They are designed for repetitive training during therapies and, thus, to perform specific movements and exert high forces. Most of these have no dimension limitations since their portability is not mandatory, even if it is still preferable, e.g., the already commercially available *Gloreha* from Idrogenet^7^ (Milia et al., 2019) or *InMotion*^®^ *ARM* from Bionick^8^. Their clinical outcomes are considered highly dependent on the mechanical design, the interaction with the patient, the adopted training mode, its duration and intensity, and the patient state (Huang et al., 2017; Zhang et al., 2018; Rodgers et al., 2019). Assistive devices are designed instead to help the users in the Activities of Daily Living (ADLs) (Sarac et al., 2019), e.g., holding a bottle of water or opening a door, by responding to their intentions. Therefore, they shall be comfortable to wear, lightweight, less bulky as possible while still exerting enough force to assist the wearer effectively. Moreover, they shall not force the hand in wrong poses and preserve the sense of touch. At the best of their capabilities, assistive HESs should allow movements as a healthy hand could make alone.

Recently, such a distinction is no longer clear-cut. Indeed, more wearable and portable devices make possible rehabilitation therapy in different environments from the clinical one (e.g., at home), reducing the burden on therapists or in-patient facilities (Huang et al., 2017). Besides, devices designed for assistance or at-home rehabilitation (Lambercy et al., 2013; Polygerinos et al., 2015; Randazzo et al., 2017; Cappello et al., 2018; Putzu et al., 2018; Wang et al., 2018; Bouteraa et al., 2019; Dittli et al., 2020; Yurkewich et al., 2020) can always be available for the patient, who may find it stimulating and motivating to perform these therapies in a home setting by playing a computer game or during typical ADLs (Butter et al., 2008; Maciejasz et al., 2014). Despite the significant results achieved, robot-assisted and home-based therapy effectiveness remains an open research topic (Maciejasz et al., 2014; Huang et al., 2017; Duret et al., 2019).

More designing aspects can also distinguish HESs. Regarding the assisted movements, the number of driven fingers—usually defining the number of independent motors, unless passive couplings between fingers are used—the Degrees Of Freedom (DOFs), and the interactions between hand and exoskeleton may differ. In particular, HESs can have a single interaction point on each finger, in the case of single-phalanx mechanisms (Dovat et al., 2008), or more than one, in multi-phalanx configurations (Tong et al., 2010; Ho et al., 2011; Lambercy et al., 2013; Cempini et al., 2014; Yun et al., 2017; Diez et al., 2018; Wang et al., 2018; Bouteraa et al., 2019; Dittli et al., 2020).

Also, different mechanical designs are commonly classified according to the strategies for placing the device on the hand and fingers, e.g., on the palm (Bouzit et al., 2002; Putzu et al., 2018), the back, as most of the presented solutions, or even involving the finger sides (Lambercy et al., 2013; Cempini et al., 2014; Yu et al., 2019). In addition, they can be rigid or soft exoskeletons. The first ones (Bouzit et al., 2002; Tong et al., 2010; Ho et al., 2011; Lambercy et al., 2013; Cempini et al., 2014; Yun et al., 2017; Diez et al., 2018; Wang et al., 2018; Bouteraa et al., 2019) are made of metal or plastic, transmit motion through rigid kinematic chains, and usually exert higher force than soft ones (Chiaradia et al., 2018; Chu and Patterson, 2018). Indeed, on the other side, these are made of flexible materials (In et al., 2015; Polygerinos et al., 2015; Randazzo et al., 2017; Cappello et al., 2018; Hadi et al., 2018; Putzu et al., 2018; Yu et al., 2019; Dittli et al., 2020; Yurkewich et al., 2020) and are smaller and lighter than rigid ones. Recently, first hybrid solutions have been studied (Rose and O'alley, 2018) aiming to exploit the strengths of both.

Hand Exoskeleton Systems also depend on the actuation system, which can be electrical (Tong et al., 2010; Ho et al., 2011; Lambercy et al., 2013; Cempini et al., 2014; Randazzo et al., 2017; Yun et al., 2017; Diez et al., 2018; Wang et al., 2018; Bouteraa et al., 2019; Dittli et al., 2020; Yurkewich et al., 2020), pneumatic (Bouzit et al., 2002; Cappello et al., 2018; Putzu et al., 2018), hydraulic (Polygerinos et al., 2015), or realized through different working principles, e.g., using shape memory alloy actuators (Hadi et al., 2018).

Finally, exoskeletons differ in the employed method and sensors for finger pose tracking during operation, e.g., optical, flex, magnetic sensors, or finger exerted forces measuring. Such devices might also be passive or active, and they might be distinguished in the way of detecting the user intentions near correctly as possible, e.g., using surface ElectroMyoGraphic (sEMG) signals (Ho et al., 2011; Meng et al., 2017; Yun et al., 2017; Rose and O'alley, 2018; Wang et al., 2018; Bouteraa et al., 2019; Dittli et al., 2020; Yurkewich et al., 2020).

Although many alternatives exist in the literature, not all of them are fully wearable and portable solutions. It is reasonable to state that the more compact, light weight, and standalone the device, the better the wearability. Furthermore, preserving the user freedom of movement and comfort is crucial for these tools to be handy. Indeed, these are critical features that profoundly affect such device characterization (Desplenter et al., 2020), allowing them to broaden their application fields. A solution that concentrates its components as much as possible on the assisted limb is preferable concerning wearability and portability to those that displace some units (e.g., actuation or power supply) to other body parts, limiting freedom of motion (Desplenter et al., 2020) [e.g., in a waist belt (Polygerinos et al., 2015), in the arm (In et al., 2015), in the back (Rose and O'alley, 2018; Dittli et al., 2020), and in the chest (Randazzo et al., 2017)]. For instance, forces exerted by soft structures might be increased by exploiting pneumatic or hydraulic actuators. However, these also augment the overall device weight, requiring different placement typically for both the actuators and control units (Polygerinos et al., 2015), thus limiting the user mobility. Significantly, a fully wearable and portable device can help the patient in ADLs and make available rehabilitation training in most places of daily life and thus also at home (Chu and Patterson, 2018; Wang et al., 2018). Depending on the patient state, this might prevent the constant therapist presence, who would nevertheless have to assess the rehabilitation progress periodically and guide the following training. It might reduce both the device and treatment cost and facilitate repetitive training (Huang et al., 2017).

Table 1 summarizes some characteristics (of interest for the focus of the discussion proposed in this study) of the leading current state-of-the-art assistive and portable rehabilitation devices and some also commercially available: the actuated fingers, wearability, exerted forces, weight on the hand, and intention detection method.

**Table 1:** The table shows the comparison between some of the most interesting assistive and rehabilitative hand exoskeletons at the current state of the art.

**Devices**	**Actuated fingers**	**Wearability**	**Exerted force [N]**	**Mass [g]**	**Intention detection method**
Ho et al. (2011)	All	/	/	500	sEMG
In et al. (2015)	Index, middle	Yes (*)	9–12	194	Wrist motion
Polygerinos et al. (2015)	All	Yes (*)	8	285	Force, position
Randazzo et al. (2017)	All	Yes (*)	5	50	EEG activity
Yun et al. (2017)	Thumb, Index, Midlle	Yes (*)	/	319	sEMG
Diez et al. (2018)	All	No	12,5	/	Force, position
Hadi et al. (2018)	All	/	8	300	/
Rose and O'alley (2018)	All	Yes (*)	40	220	sEMG
Wang et al. (2018)	All	/	/	420	sEMG, voice control
Bouteraa et al. (2019)	All	Yes	/	388	sEMG
Yu et al. (2019)	All	No	22	300	/
Dittli et al. (2020)	All except for the thumb	Yes (*)	/	113	sEMG
Yurkewich et al. (2020)	All	Yes	/	377	sEMG
Carbonhand	Thumb, middle, ring	Yes (*)	5–8	85	Force
exomotion®	All	Yes (*)	/	/	Impulses of an active muscle
HandyRehab	All	Yes	/	380	sEMG
Neomano	Thumb, index, middle	Yes (*)	20	105	/

(*) some of the parts of the HES—e.g., the power supply or actuation system —are dislocated from the hand.

The research activities of the Mechatronics and Dynamic Modeling Laboratory (MDM Lab) at the Department of Industrial Engineering (DIEF) at the University of Florence (UNIFI) have been focusing on wearable devices since 2013. The research team has developed several versions of a HES (Secciani et al., 2018), whose primary purpose is to assist users in ADLs. More strict requirements for the assistance aim led to this choice. However, such a device can also be used for rehabilitative purposes. Indeed, some preliminary tests on the patient with the HES have been carried out in a clinical setting, like a rehabilitation session, proving the device effectiveness in assisting the user in ADLs (Secciani et al., 2019). Despite promising results, the developed prototypes all presented gaps that did not allow them to be fully wearable solutions.

Conversely, the version (Secciani et al., 2021), presented in detail here, embodies the evolution of the previous designs to overcome such wearability limits. It is essential to point out that no performance improvements have been implemented from the last version, but instead, they remain similar, mainly because of exploiting the same actuator and kinematic structure. For these reasons, the main contribution of the work reported in this study relies on the innovative mechatronic design that results in a fully wearable and portable robotic device for assisting impaired hands. In contrast to most other state-of-the-art devices, it is particularly noteworthy that the solution presented here eliminates components dislocation, maximizing the exoskeleton wearability. *Action research arm test (ARAT)* has been performed to prove the HES capabilities and evaluate its redesign pros and cons.

This study is organized as described below. In section 2, the main strengths and flaws of the previous prototypes will be highlighted, laying the foundations for the further development presented in this study. Section 3 will present the changes made to solve the mentioned problems. In section 4, *ARAT* and its results will be presented. The strengths and flaws of the achieved solution will be discussed in detail in Section 5, based on the conducted tests. Finally, section 6 will conclude the this study.

## 2. The Previous Prototypes: Strengths and Flaws

This research activity has always aimed to develop an easily wearable, small, lightweight, safe, and low-cost robotic device for users with impaired hands. The HES prototype presented in detail in this study results from the evolution of three previous versions (Secciani et al., 2018). The overall architecture key points have had no changes: It has always been based on single-phalanx, single-DOF, rigid, and cable-driven finger mechanisms, acting on all the finger, except for the thumb. Each mechanism end-effectors are on the matching fingers distal phalanx. In addition, these mechanisms have been designed: (i) to be 3D-printed in Acrylonitrile Butadiene Styrene (ABS)—chosen due to its mechanical characteristics and its lightness—; (ii) to withstand forces up to 15 N on the contact point of each matching finger, which has been proved to be a reasonable value for typical manipulation tasks of ADLs (Riddle et al., 2020).

**Figure 1:**
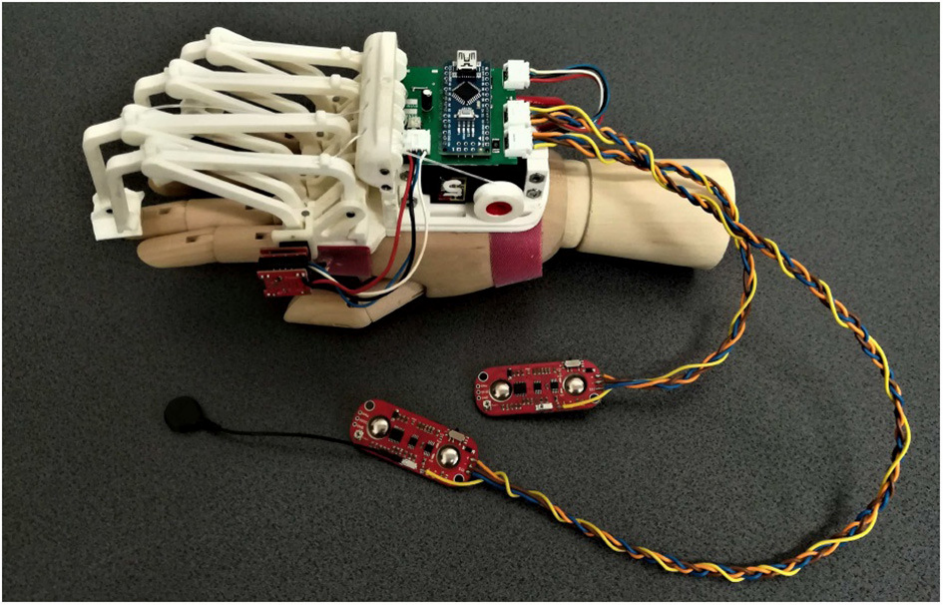
The figure shows the last version of the exoskeleton prototype before the changes proposed below in this paper (Secciani et al., 2021). Specifically, the following components can be seen: (i) four planar finger mechanisms on the left; (ii) a magnetic encoder, placed upon the mechanism joint above the index finger MCP one; (iii) two sEMG sensors, the two red boards on the bottom, and cables for data transmission; (iv) a micro-controller on a green printed circuit board, in the middle top of the figure; (v) a servomotor, the black component below the micro-controller.

The last of the previous prototypes (the third one), shown in Figure 1, has been developed focusing on patient needs, aiming to have a portable, wearable, and easily customizable device for assistive and rehabilitative purposes.

The single-DOF and single-phalanx finger-handling mechanism—made lighter and less bulky without influencing the already validated kinematic model (Conti et al., 2016)—allows reproducing the complex hand kinematics using a more compact device than most of the other state-of-the-art rigid mechanisms (Lambercy et al., 2013; Yun et al., 2017; Diez et al., 2018; Bouteraa et al., 2019). For this reason, no other cumbersome components have been added. Only a second passive DOF per finger has been added to allow ab/adduction movements, enabling the auto-alignment between fingers and their corresponding mechanisms. An optimization procedure (Bianchi et al., 2018) has been employed so that its final geometry results to be effectively tailor-made on the patient anatomy. Customization and ab/adduction movements improve ergonomics and user comfort, avoiding constraining feelings and helping the fingers more efficiently arrange during object grasping. Such considerations allow complying with some of the crucial requirements cited in section 1 (Sarac et al., 2019).

The exploitation of a single servomotor (HS-5495BH High-Torque Servo from Hitec), unlike the solutions in Table 1, is another vital topic to be considered in depth. On one side, this choice positively impacts the mechanical and electronic hardware architecture since the system weight is unavoidably lower by reducing its components. Also, the control code is more straightforward, not managing the synchronized motion of more actuators. As an inevitable drawback, the independent motion of the fingers is not allowed. However, being the finger-handling mechanisms cable-driven, the grasp shape results to be deformable to different object shapes. Indeed, the patient can perform irregular grasps since the fingers can adapt—while remaining within their own *Range Of Motion*—to the objects thanks to the cable flexibility. Nevertheless, independently controlled fingers make a significant difference only with tasks involving the tip and tripod grasps, not frequently used in essential ADLs (Montagnani et al., 2016).

By powering the servomotor with 7.4 V, it can output a maximum torque of 0.735 Nm and a maximum angular speed of 6.67 rad/s; such performances have been preliminary considered and then verified suitable to exert the forces the exoskeleton has been designed for (15 N as mentioned above), which are comparable, if not better, to the ones of the listed solutions in Table 1.

All the electronic components have been chosen to have a lighter, cheaper, and more intuitive device. An Arduino Nano controller board, a Bluetooth module, and a driver have been integrated on a custom Printed Circuit Board (PCB) (Secciani et al., 2019) and then placed on the user hand back. A magnetic encoder collects the angular position and velocity of the mechanism joint—to which it is applied—placed right above the index finger MCP joint, not preventing any hand function. The mechanism kinematics is solved as a function of this mechanism joint angular coordinate, which depends on the index finger's MCP joint flexion/extension angle. Such measurements are consistent also with the other fingers since they are all moved simultaneously by the HES. Two MyoWare^*TM*^ Muscle Sensors from Advancer Technologies (United States), low-powered devices, have been chosen to collect epidermal EMG signals, namely sEMG signals. They can detect, interpret, and measure bioelectric signals from muscle activity. Such sensors incorporate the housing for two monopolar snap electrodes into a small breakout board (20.8 × 52.3 mm^2^). Despite this, the MyoWare behaves as a single bipolar sensor capable of generating two distinct differential output types. Specifically, they represent a good trade-off between the high cost of many of these sensors and problems connected with the dedicated software for managing them.

The strategy proposed (Secciani et al., 2019) for controlling this device enables intuitive management of its motion. Indeed, it is based on the user intention classification starting from myoelectric readings. The user muscular activity is measured 50 times per second, and it is translated into a command for the actuator to follow the captured intention. Such commands can be “hand opening,” “hand closing,” and “resting.” It is worth noting that the forearm muscle closeness and sEMG signal nature and noise level require high computational power machines to classify user movements accurately. Classifying just three elementary intentions has resulted in a reasonable trade-off between complexity and usability. The first two intentions imply an effective system motion, while the third represents a security state for which the device remains in its current position. The motor velocity is set to perform a complete opening gesture in about 1 s; the same applies to complete closure.

A custom Graphical User Interface (GUI) has been developed to (i) collect sEMG signals training datasets by recording them, (ii) manually draw two polygons around the points that identify the opening and closing gesture, (iii) upload the classifier parameters to the micro-controller board, and (iv) save the polygons vertices coordinates (Secciani et al., 2020). By doing so, opening or closing intentions are classified according to the polygon the corresponding points belong to. Those points that belong to both the polygons or none of them are classified as a rest intention.

Another GUI has been developed for an intermediate training phase for both the classifier and user. A 3D hand model is displayed, and it moves according to the user intention classification. The interface allows controlling the complete hand opening and closing, and the intermediate positions, helping the user to get used to the HES and classifier. From the user point of view, straightforward device managing is one of the main goals of this version.

At this point of the study, the HES is customizable on the patient hand, compliant with it, and intuitive, thanks to the user intention detection method. Nevertheless, some flaws have remained, especially non-full device wearability and portability. During a preliminary testing phase, some criticisms in cable management and dressing the electromyographic sensors have arisen and impose severe limitations on the device use. Such tests have been carried out in the laboratory and only on healthy subjects to assess the new integrated electronics. The subjects were required to wear the MyoWare^*TM*^ sensors on the forearm and trigger the servomotor motion with muscle contractions. It was observed that wrist movements could occasionally produce excessive tensions in cables connecting the sEMG sensors to the microcontroller (see Figure 1). Besides, these cables elasticity caused small shifts between the sensor and user skin. These displacements compromised the signal acquisition and were then translated into the so-called “motion artifacts,” which resulted in erroneous intention classifications. Therefore, cables represented a hindrance and another annoyance for the patient, preventing the HES from complete portability.

Also, the connection between the hand and exoskeleton represented a crucial issue to be solved. Such an interface was produced by sewing the device to a sports glove and then fixing it to the limb with additional Velcro bands, as in Tong et al. (2010), Ho et al. (2011), Lambercy et al. (2013), Rose and O'alley (2018), and Wang et al. (2018). However, on one side, elasticity of both these systems ensured high-grade coupling safety because of their intrinsic compliance with any possible displacement. On the other side, the same feature could cause an inconsistency between the exoskeleton actual trajectories and the fingers ones it has been designed for. Indeed, the exoskeleton motion may produce a change in its relative position to the back of the hand, not ensuring the same movements reliable repeatability.

Finally, the lack of an on-board power supply system, as also in In et al. (2015), Polygerinos et al. (2015), Randazzo et al. (2017), Diez et al. (2018), Rose and O'alley (2018), Yu et al. (2019), and Dittli et al. (2020) or in *exomotion*^®^*, Carbonhand* and *Neomano* devices among the commercially available ones, as visible in Table 1, prevented the device from being fully wearable and portable. Being an exoskeleton intended for assistive use, as already mentioned, this point was one of the most limiting since it forced the user to be connected to a power supply away from the hand.

## 3. The New Architecture

This section will present the changes made to overcome the above-mentioned issues and develop a fully wearable device. Specifically, the renewed sEMG technology, the improved ergonomics of the interface between the hand and exoskeleton, and the revamped mechatronic architecture are presented.

### 3.1. Surface Electromyography Technology

**Figure 2:**
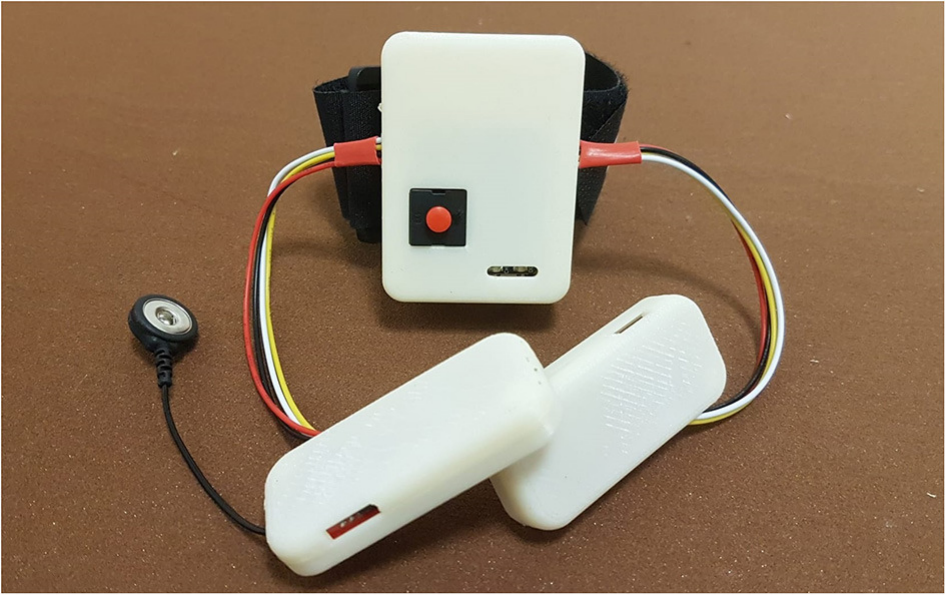
The figure shows the sEMG bracelet, developed to reduced the disturbances coming from the sensor wires, with its central unit and the other two smaller ones containing the sEMG sensors.

Among the several commercially available sensors, which are usually exploited to interpret the myoelectric activity (Rechy-Ramirez and Hu, 2015), sEMG sensors have also been employed in this new device version due to their capability of detecting electromyographic signal directly through the skin, as a completely non-invasive technique. Some preliminary tests have been carried out (Secciani et al., 2019), proving that the device effectively assists patients with muscular (rather than neuronal) deficits in ADLs. Indeed, as long as the user can emit controlled myoelectric signals—even if weak—the system can sample them, classify them, and then replace the musculoskeletal system. For these reasons, such a HES can be used in all cases, acute or chronic, in which the disability does not compromise the user ability to associate a motion intention with a specific muscle activation voluntarily. For this kind of application, the sEMG sensors chosen are usually placed on the antagonist muscle bands responsible for the fingers and wrist flexion/extension, which are on the *extensor digitorum* and *flexor digitorum* muscles. A first tentative bracelet for sEMG signals collection has been designed to overcome cable issues presented in section 2 and mitigate the disturbances introduced. It has been thought to be worn on the forearm. The result is shown in Figure 2. As visible, the sEMG bracelet consists of three cases that are 3D-printed in ABS. The central unit, the biggest one, has been thought to house the microcontroller, the Bluetooth module, and a single-cell 500 mAh Li-ion battery, while the other two hold only the sEMG sensors. Each wire between the central unit and the two sensor housings has a different function following its color: The red one is the 3.3 V line, the black one represents the ground line, sEMG signals flow in the yellow wire, while the white one represents the reference potential. Thus, the long cables, shown in Figure 1, connecting the sensors directly to the exoskeleton actuation system, have been removed. The servomotor command signals are now processed on-board and then sent to it thanks to the micro-controller on the exoskeleton over a Bluetooth bridge. The resulting total weight of the bracelet is around 80 g.

### 3.2. Ergonomics

**Figure 3:**
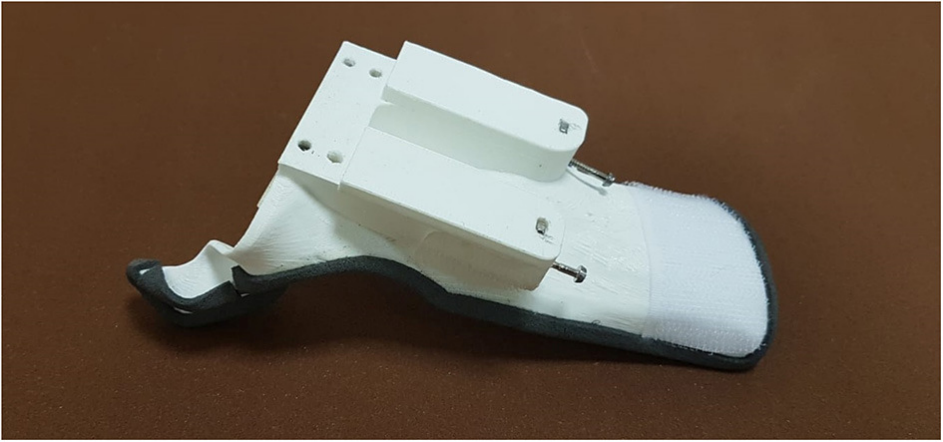
The figure shows the 3D-printed anatomical splint with the slide and the holes for mounting the two HES modules.

The connection between the hand and exoskeleton has been improved to make the device more comfortable for the wearer, avoiding the issues mentioned above about motion accuracy. It has been achieved by exploiting an anatomical wrist splint, which provides a sufficient rigid interface base with the forearm. A splint is meant as a device that increases, improves, or controls an impaired function of an injured segment, e.g., such a tool is usually used to support a broken bone and keep it in a fixed position or during rehabilitation treatments. It is commonly made of a thermoplastic material mouldable at relatively low temperatures (about 75°), customized and modeled by a therapist directly on the patient anatomy. Its main features are being lightweight, resistant, easy to wear and remove, washable, and continuously adaptable to the evolving patient needs. However, such a thermoformed splint does not have a support base to anchor the exoskeleton. So, a ROMER arm equipped with a 3D laser scanner—a completely portable coordinate measuring machine—has been used to acquire the splint surfaces and produce a considerable number of point clouds to create a 3D CAD model. After collecting sufficient data, the point clouds have been cleaned and smoothed using Polyworks and Geomagic Design X software. After that, the points have been triangulated, aligned, and finally merged in one only surface. The splint 3D CAD model is rebuilt starting from this optimized surface, and, by doing so, its lower surface shape accurately reproduces the forearm anatomy. Then, such a model has been modified as shown in Figure 3: The fingers module housing, in which there are four threaded holes for the screws employed for their attachment, and a magnetic slide for fixing the motor box has been designed. Finally, the splint has been 3D-printed in PolyLactic Acid (PLA), resulting in a thickness of 2 mm and a weight of only 42 g. A stable interface between the hand and HES is then provided by connecting it with the exoskeleton. The connection, removable whenever needed, is achieved by exploiting four screws for the finger mechanisms module, the slide, and two other screws to fix the motor box to the new splint. Among the possible functionalities of a splint, this tool employed as a part of the new prototype and presented in this study (see Figure 3) can be identified as an integrative splint—since it allows to compensate compromised limb functions—and one for protected mobilization—since it aims to improve specific muscle activity. Indeed, on one side, it enables to stiffen the wrist articulation so that the interaction forces between the hand and exoskeleton are spread throughout the forearm, concentrating the HES action on the fingers and giving better stability to the system. On the other side, it also provides rigid support for the thumb to keep its first phalanx in a semi-opposition state to the palm, thus facilitating object grasping by mitigating tendon retraction. Finally, even in this case, the HES can be fixed on the hand using Velcro bands, providing the interface with a bit of compliance for improved wearer comfort.

### 3.3. Overall Architecture and Power Supply System

The last and most significant changes have been made to the overall mechatronic structure, revamping the power supply and transmission system to achieve the complete portability of the device.

**Figure 4:**
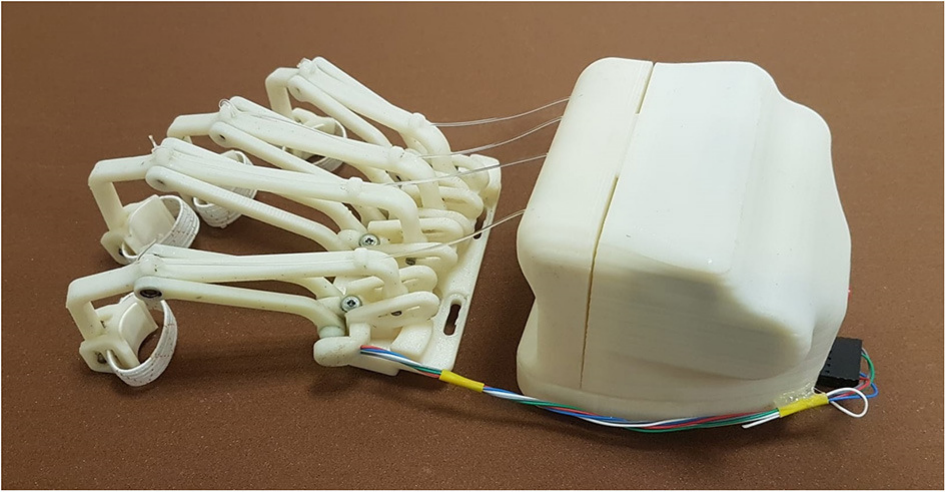
The figure shows the two blocks of the HES modular structure: on the left, the fingers' mechanisms module, and on the right, the motor and control one.

The first crucial difference of this new HES version is the whole system modular structure, which is now essentially divided into two blocks, shown in Figure 4: The one including the motor and control box is placed on the hand back, while the one that houses all the finger mechanisms is located right above the fingers.

Such a structure has been designed to minimize downtime for maintenance, whether it is programmed or not. While the back module may be subjected to sporadic changes, e.g., a smaller one may be required for a pediatric exoskeleton (Bianchi et al., 2019), the front one will be more subject to replacements due to a mobility recovery or, on the contrary, a pathology evolution. Indeed, the finger trajectories may change, and thus, their mechanisms geometry might need to be revised. So, the fingers module is connected with the 3D-printed splint thanks to four screws, easily removable. In addition, as visible in Figure 4, four slots are realized in this module to help regulate its position and match each mechanism with its corresponding finger correctly. The two modules total mass on the hand is 415 g.

**Figure 5:**
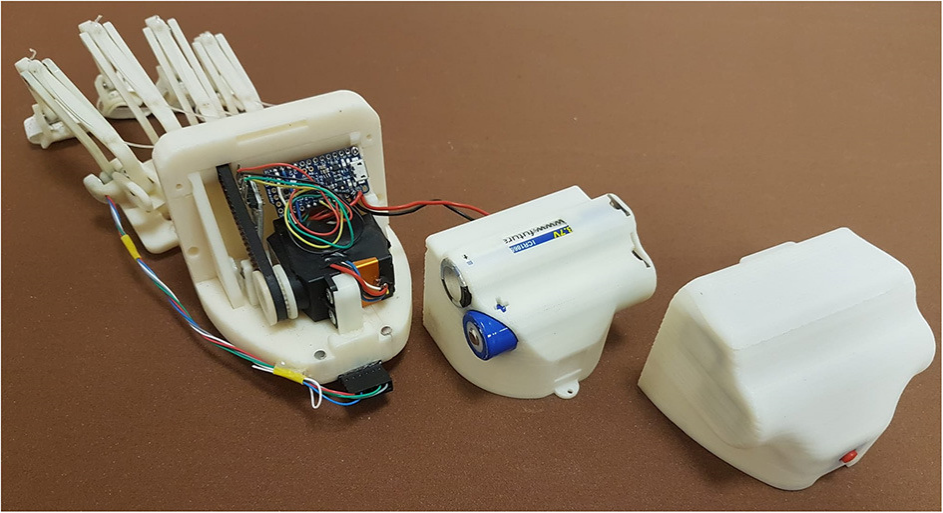
The figure shows an exploded view of the new motor box and the three layers that compose it: on the left, the inner layer, including the transmission system, actuator, and control electronics; on the centre, the middle one containing the batteries, and, on the right, the outer one, including the switch button.

Another variation to the previous version is about the power supply system (see Figure 5). It is now incorporated into the electronics case, and thus, it is no longer part of external equipment but directly integrated on the hand back. This module has been modified to contain the following components: the actuator, transmission system, control electronics, batteries, and a switch button. All these elements are disposed on three different layers, as shown in Figure 5: The actuator, transmission system, and control electronics are into the inner one; the batteries on the middle; and the switch button on the outer one. The layer on the middle is the last externally accessible to let the user change the batteries. Such a new power supply system consists of two rechargeable 3.7 V lithium-ion batteries with 2,600 mAh capacity serially connected.

Third, also the actuation system has been subject to change. Motors have been reduced from four (on the first HES prototype) to one, resulting in an unavoidable redesign of the finger mechanisms motion transmission and, thus, the overall actuation module. Indeed, a specific cable-driven transmission has been developed: Four pulleys with different diameters, depending on the fingers dimensions, have been designed and embedded to a single secondary shaft to obtain the same angular speed for all the fingers. Instead of cable that wraps and unwinds around the pulley integral with the motor shaft, a toothed belt drive is now used so that the motor sets in motion the shaft to which the four pulleys are integral. This adjustment allows obtaining the same angular velocity for all the fingers as for the previous versions, even though their trajectories involve different cable lengths.

Finally, it is worth highlighting that, as in the previous versions, additional force sensing or actuators, e.g., Series Elastic Actuators (SEA) (Yun et al., 2017) or Force Sensitive Resistor (FSR), have not been exploited to avoid additional components that increase overall complexity, dimensions, and weight, thus limiting the portability and wearability. For this reason, the proposed HES operates only in position and speed control modes, while force control mode has not been currently implemented.

### 3.4. Hand Exoskeleton System Development and Use

So far, the changes to overcome the primary limits of the previous version and achieve a fully wearable and portable device have been presented. This subsection summarizes instead the main steps required to actually develop such a customized hand exoskeleton. Firstly, the metacarpal bones and phalanges lengths are required to solve the hand kinematics and find the trajectory of the desired fingers. Such trajectories becomes then the inputs for the optimization procedure that will calculate the link dimensions of each finger mechanism. Finger-handling mechanisms are hence customized on the patient anatomy and follow the corresponding desired trajectory as accurately as possible. Once the 3D parametric model of the whole HES has been updated in the CAD software, all the components can be 3D-printed and then assembled. While finger mechanisms change from patient to patient, the actuation and control box usually remains the same, and this is why the system is designed to be split into two principal parts as described in subsection 3.3. In parallel, an anatomical splint is developed starting from a 3D scan of the user limb and then 3D-printed. The splint becomes the main interface with the hand, and the whole system is rigidly fixed to it. Elastic rings are used to fix the device to the fingers, while the splint is fixed to the hand and forearm with velcro bands. Finally, to control the HES motion, the user has to wear the sEMG bracelet and perform a preliminary phase of system training. Such step consists of a repetition of elementary muscle contractions (i.e., opening, closing, and resisting) to match with a specific exoskeleton action (i.e., pulling cables, releasing cables, and idling). Once the classifier is properly trained, the HES is ready to be used.

The kinds of patient for whom the exoskeleton is suited for are all those who can arbitrarily contract the muscles, as it is the only way to control the motion. Besides, the device, in its current status, is capable of assisting only people with hand opening impairments.

## 4. Test and Results

Experimental tests have been carried out to assess the new redesigned HES capabilities. The tested exoskeleton is tailor-made for a patient's hand, who has followed this research from the beginning. The subject is affected by Spinal Muscular Atrophy (SMA) type II since birth. Such a disease damaged muscular extensors of both his hands, causing their opening impairment due to tendon retroactions, and therefore, now hands are closed like fists. The tests have been performed to evaluate the HES actual effectiveness for the pilot study patient and whether improvements have been made after redesigning it, as presented in section 3. Specifically, they are used to understand whether the new HES exploitation can improve the patient assistance in ADLs, enabling him to grasp objects more effectively than when the device is not worn, if he has some advantages or disadvantages in using it, its new strengths, and remaining flaws. ARAT (De Weerdt and Harrison, 1985; Yozbatiran et al., 2008) has been conducted for such experimental sessions since it evaluates grasp, grip, pinch, and gross arm movements, usual in daily life activities (Duruöz, 2014). It is a functional evaluation test that assesses the upper limb functions. The test takes approximately 5–15 min to administer and requires standardized equipment: various sized blocks of wood, cricket ball, stone, glasses, tubes, washer and bolt, ball bearing, and marble. Standard protocol requires the patient to be seated in a chair facing a table, with the head in a neutral position and feet on the floor. The test is organized into four subgroups corresponding to the four different motions evaluated, with 19 items presented. Each item must be grasped and lifted on a 37-cm-high shelf above the table facing the subject.

The patient performance is rated on a 4-point scale, ranging from 0 (no movement possible) to 3 (movement correctly performed). The maximum obtainable score for ARAT is 57. Each item in the four subgroups has a well-standardized presentation order: First, the patient is asked to manipulate the most challenging object of the considered subgroup. If the task is correctly performed, enabling a total score, he is credited with having scored 3 on all the remaining subtest items without performing them. However, if the patient fails the first task and scores less than 3, the most manageable object is tested. If unlikely the patient scores 0, the remaining subtest is credited with 0, and the evaluation proceeds to the following subgroup. If otherwise, the patient scores more than 0, all items in the subtest should be assessed. The standard protocol indicates that each task might run up to 60 s if the patient does not complete it before. Specifically, the items in each subgroup are shown in Table 2.

**Table 2:** The table shows the ARAT items.

	G1	Block, wood, 10 cm cube (most difficult)
	G2	Block, wood, 2,5 cm cube (easiest)
Grasp	G3	Block, wood, 5 cm cube
	G4	Block, wood, 7,5 cm cube
	G5	Ball (Cricket), 7,5 cm diameter
	G6	Stone 10 x 2,5 x 1 cm
	GR1	Pour water from glass to glass (most difficult)
	GR2	Tube 2,25 cm (easiest)
Grip	GR3	Tube 1 x 16 cm
	GR4	Washer (3,5 cm diameter) over bolt
	P1	Ball bearing, 6 mm, 3rd finger and thumb (most difficult)
	P2	Marble, 1,5 cm, index finger and thumb (easiest)
Pinch	P3	Ball bearing 2nd finger and thumb
	P4	Ball bearing 1st finger and thumb
	P5	Marble 3rd finger and thumb
	P6	Marble 2nd finger and thumb
	GM1	Place hand behind head
Gross movements	GM2	Place hand on top of head
	GM3	Hand to mouth

*The first column includes the four subgroups. In the second one, there are direct identifiers to each task: letters indicate the corresponding subgroup, while numbers show the order in which the tasks are proposed to the subject. The third column describes the activity explicitly to perform.*

For this case study, experimental tests have been carried out in a clinical environment, and the patient was seated in his wheelchair facing the table. The tests have been repeated three times. In the first session, the patient carried out the test without wearing the HES, while he had to wear it during the second and third ones. The second session has been conducted considering a motor speed of 4,000 counts/s, enabling a complete closing in 1.2 s. The same applies to the complete opening. The third session has been instead performed at a 50%-increased motor speed (6,000 counts/s, allowing a complete closing/opening in 0.8 s) after finding that it did not cause discomfort to the patient.

**Figure 6:**
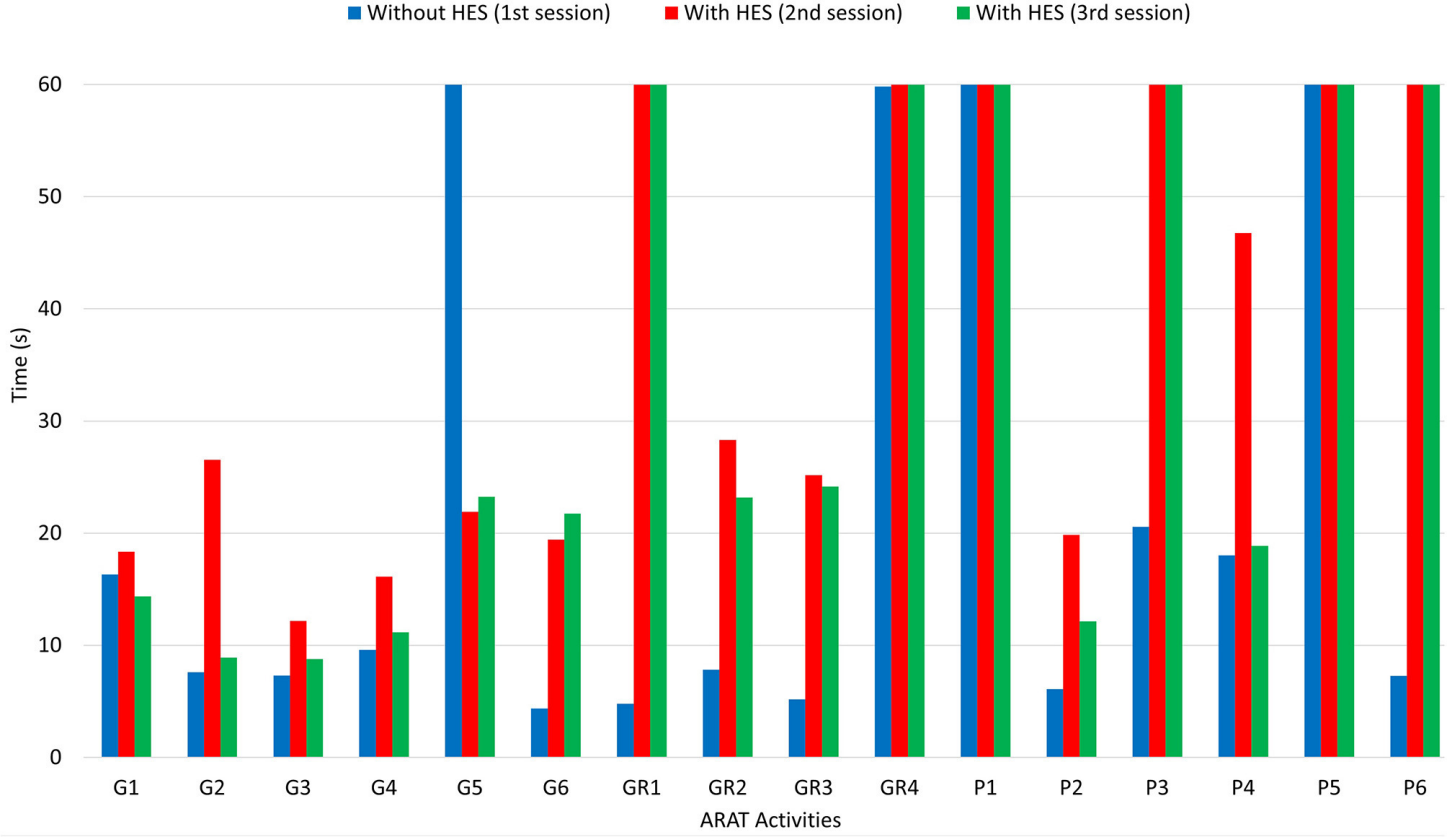
The figure shows a histogram concerning the results of the ARAT carried out. While activities are reported in the horizontal axis, the time (in seconds) needed for each one corresponds to the height of the column, readable in the vertical axis.

After carrying out the first session and taking the time to perform each ARAT task, a physiotherapist helped the patient donning the sEMG bracelet and HES on the right upper limb. The two sEMG sensors have to be placed as described in subsection 3.1. The donning phase and sensor placement required about 5 min since particular attention must be paid to avoid painful movements and find the correct spot for sensors. In such a case, it has been necessary to consider the wrist muscular activity due to the difficulty of providing strong finger extension signals without heavy fatigue. The first GUI presented in section 2 has been exploited to collect sEMG signals training datasets, draw the corresponding opening and closing gestures polygons, and upload the classifier to distinguish each intention. Instead, the second one is employed to have an intermediate training phase for the patient wearing the HES. Then, the interfaces have been closed, and the other two sessions started. Table 3 shows the scores the physiotherapist gave to each task. It is possible to understand that the patient has no arm-motion impairments, having scored 3 points for gross movements (GM1, GM2, and GM3) wearing the HES or not. Instead, in all the other tasks, more or fewer difficulties have been found. Thus, significantly, the results concerning only the first three subgroups (G, GR, and P) are also shown in terms of time thanks to a histogram (see Figure 6), and they are reported for all three sessions. The histogram indicates the time (in the vertical axis) recorded to complete each task (specified instead in the horizontal axis) during different session settings. Specifically, the blue column height shows the time taken to complete the task without the HES, the red column height the time taken wearing the HES with a motor speed of 4,000 counts/s, while the green one refers to the session in which the motor speed is increased up to 6,000 counts/s. Sixty seconds has have been awarded to the tasks the patient failed to complete.

**Table 3:** The table shows the scores obtained during the three sessions of ARAT.

**Sessions**	**G1**	**G2**	**G3**	**G4**	**G5**	**G6**	**GR1**	**GR2**	**GR3**	**GR4**	**P1**	**P2**	**P3**	**P4**	**P5**	**P6**	**GM1**	**GM2**	**GM3**	**Total score**
Without HES	2	2	2	2	1	2	2	2	2	2	0	2	2	2	0	2	3	3	3	36
With HES	2	2	2	2	2	2	0	2	2	0	0	2	0	2	0	0	3	3	3	29
With HES	2	2	2	2	2	2	0	2	2	0	0	2	0	2	0	0	3	3	3	29

**Figure 7:**
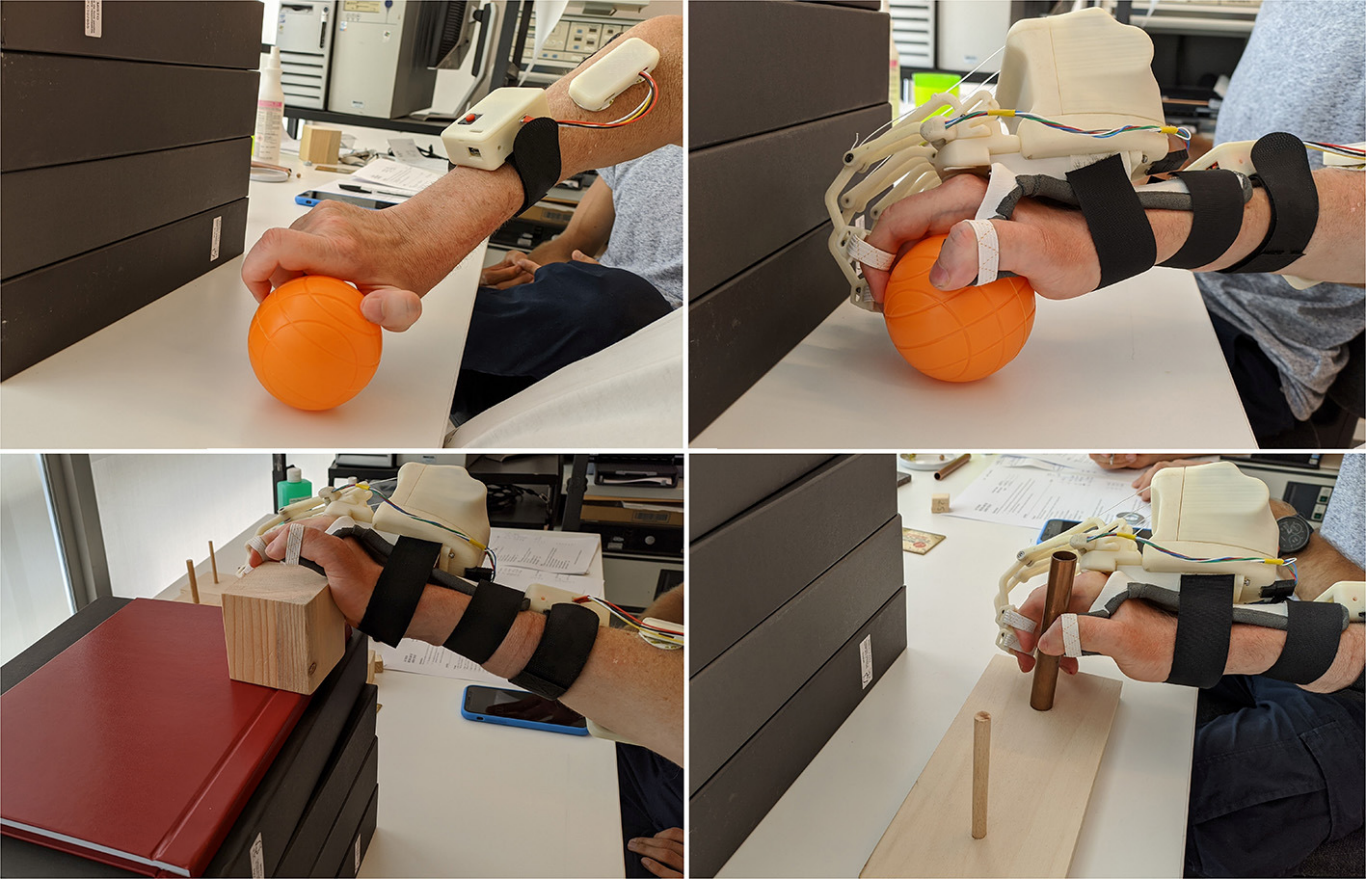
The figure shows some pictures taken during the test: (i) on the top-left, the cricket ball grasping (G5) performed without the HES, (ii) on the top-right, the same task performed with the HES, (iii) on the bottom-left, the wooden block grasping (G1) is shown, while (iv) on the bottom-right, the alloy tube gripping (GR2) is visible.

The histogram shows that the times taken for grasping activities during the first and third sessions are comparable. Instead, longer times have been recorded during the second one. Besides, the HES exploitation allowed better-grasping objects. For instance, Figure 7 shows the 7.5-cm-diameter cricket ball grasping (G5) without the HES (on the top-left) and wearing it (on the top-right). It is possible to observe that without the HES, the patient has adjusted his grasping according to the movements he can perform. On the contrary, the HES enabled the patient to fully open the hand and grasp the ball correctly and effectively. The same happened for the 10-cm-cube wooden block grasping (G1), visible in Figure 7 bottom-left.

More difficulties have been found among grip movements wearing the HES on glasses (GR1) and washer (GR4) manipulation. Significantly, the washer is highly challenging to grip for the patient also without the HES due to its shallow thickness. Conversely, alloy tubes grips (GR2 and GR3) have been performed without the HES and wearing it, as visible in Figure 7 bottom-right. The time taken on the second and third sessions is more than the one on the first, but also now, the HES enabled the patient to have a more correct and effective hand grip.

Finally, from Figure 6, it is possible to observe that the patient has found more impairments on pinch movements both with and without the HES. Indeed, he could not perform two out of six pinch tasks during the first session, but he failed four out of six tasks during the second and third. The augmented velocity on the third session enabled times comparable with those recorded without the HES, even if they are still greater. The worsening performances are due to the overall dimensions of the finger-handling mechanisms, which, although they have been reduced compared to the first prototype ones, as visible in Table 4, still prevent movements in confined spaces, e.g., when the patient hand approaches the table to pinch small-size items on it. Specifically, he did not fail the task in which index and thumb fingers had to interact. Instead, when the patient had to pinch items with the middle or ring finger and thumb, such impairment did not allow him to complete the tasks.

**Table 4:** The table compares the overall lengths and heights of the first, and the one proposed here prototypes finger mechanisms.

**Fingers**	**First HES**		**New HES**	
	**Length [mm]**	**Height [mm]**	**Length [mm]**	**Height [mm]**
Index	98,42	35	85,61	35,13
Middle	107,6	48	95,44	39,17
Ring	100	36	87,31	35,84
Small	74	27	71,91	29,51

## 5. Discussion

In this section, the new exoskeleton prototype strengths and weaknesses will now be discussed. The main focus of this study was the renewal of the system architecture to achieve a fully wearable, portable, comfortable, and tailor-made robotic device for home assistance in ADLs and telerehabilitation. The experimental tests presented in section 4 proved that such an aim had been correctly pursued. Indeed, the redesigned HES is now fully wearable and portable, and so it was for the patient throughout the tests—which lasted about 3 h, including the starting training phase and some resting breaks. The patient could perform all the sessions without much discomfort, even if he still fails some tasks. Such failures are primarily due to the HES overall dimensions that prevent tasks in confined spaces. Besides, the patient needs a more extended training period with the HES to get used to it and how the device detects his intentions. Specifically, the redesigned device enables the user to open the hand starting from a fist and then perform the grasps correctly. It also allows the patient to hold the item due to its force effectiveness until he wants to release it, and a hand opening intention detected from the sEMG sensors causes the cables to wind up.

Below, direct references to the three subsections above will be made to discuss the improvements presented on each.

**sEMG technology (subsection** **3.1)**-The exploitation of sEMG sensors enables the HES control system to collect and interpret signals from muscular activity in a completely non-invasive way. Various solutions from Table 1 exploit sEMG signals to detect the user intention (Ho et al., 2011; Yun et al., 2017; Rose and O'alley, 2018; Wang et al., 2018; Bouteraa et al., 2019; Dittli et al., 2020; Yurkewich et al., 2020), but only in Rose and O'alley (2018) and Yurkewich et al. (2020) and *HandyRehab* a bracelet, are exploited to collect such signals. The sEMG bracelet proposed in this study represents one more step toward an entirely intuitive device, free from cumbersome cables and external equipment. The advantages of such a solution are not limited to the disturbance reduction, which is achieved by physically decoupling the acquisition system from the wrist and exploiting a Bluetooth bridge for data transmission. Indeed, it has also been proved to help lighten the microcontroller workload on the exoskeleton, which is no longer slowed down by the sEMG signals sampling, preprocessing, and classification. It is worth highlighting that the Bluetooth bridge avoids cables presence and enables the system to stream sEMG data to any external platform for development and monitoring purposes, even remotely. The experimental tests conducted (and presented in the previous section) have proved that the bracelet improved the HES, being comfortably portable and effortlessly wearable, mitigating the patient feeling of constraint on the forearm due to cables.

**Ergonomics (subsection** **3.2)**-An ergonomic mechanical design of a wearable assistive and rehabilitative device should guarantee kinematic compatibility with the user fingers and a comfortable mechanical-physical interface (Chiri et al., 2012). The new prototype ergonomics have been increased thanks to a tailor-made PLA splint that provides a high-stability kinematic coupling with the user limb. This feature allows the mechanisms to follow the desired finger trajectory correctly with decent repeatability. This aspect is extremely crucial to prevent the exoskeleton from forcing the hand into wrong and painful poses. Specifically, the splint avoids the poor stability that was due to the glove elasticity and ensures a better distribution of efforts on the hand and forearm. Before starting tests, 1-mm-thick Neoprene adhesive strips have been added to the splint inner surface and edges to reduce direct contact with the skin and the consequent skin redness and irritation. Only a commercially available solution, the *exomotion*^®^, exploits the Reverse Engineering to achieve a glove and an arm splint designed for the patient, to the best of the authors' knowledge. Its plaster casts are used in this case to mold a glove, which has to be scanned and then rebuilt. Instead, for many solutions, a simple platform to hold all the components is designed (Lambercy et al., 2013; Diez et al., 2018; Wang et al., 2018; Dittli et al., 2020). Only occasionally, it is curved according to the natural hand profile but still not tailor-made on the specific patient hand (Tong et al., 2010; Ho et al., 2011).

Although improved over previous versions, the ergonomics of the device still have plenty of room for improvement. Firstly, the splint might be improved by exploiting a reticular structure with variable stiffness, making it more breathable and fitting to the hand. Second, the development of a donning/doffing system to let the patient autonomously wear the exoskeleton would be crucial. To the best of the authors' knowledge, there are no devices that include such a system in the literature. In its current state, this solution is only a first step toward a system fully compliant with the hand, which can provide ergonomic support to fix the exoskeleton. Also, the splint makes the HES donning/doffing phase easier, reducing the time the therapist spent on this procedure while guaranteeing noteworthy kinematic stability.

**Overall architecture and power supply system (subsection** **3.3)**-The most significant innovation of this new architecture compared to the current state-of-the-art wearable robots—at least to the best of the authors' knowledge—is undoubtedly owed to this aspect. The exoskeleton is now standalone and entirely wearable. The modular structure is differently proposed in some other devices (In et al., 2015; Polygerinos et al., 2015; Randazzo et al., 2017; Hadi et al., 2018; Rose and O'alley, 2018; Yu et al., 2019; Dittli et al., 2020), also in the commercially available ones, to enable some components dislocation and lightening the device on the hand back, against complete wearability and portability. The proposed solution makes faster and more streamlined the HES design process, its embodiment, its assembly, and its regulation on the hand, while not foreclosing the complete wearability and portability features instead. Besides, such a structure reduces the maintenance times since only the modules eventually needed can be replaced.

Toothed belt exploitation between the motor and secondary shaft makes the motion transmission more accurate and stable, preventing unexpected cable unwinding, which could cause errors during hand opening or closing. The total encapsulation of the power supply system and the electronics (including the one for processing sEMG signals) prevents the device from exposing delicate components—which otherwise should have to be placed along the upper-limb—resulting in a smaller and electrically safer design, thus not constraining the patient movements. Indeed, these changes allow achieving the following overall dimensions of the module on the hand back: 80 mm in width, 72.5 mm in length, and 70.6 mm in height. In addition, the new mechanisms bulkiness is smaller than that of the previous prototype, as visible in Table 4, by streamlining the structure. It is worth noting that all the length sizes have been reduced, having removed some components. Instead, the heights have remained relatively similar to the previous one. However, such a solution results also in a worse masses distribution. Indeed, about 460 g is now concentrated on the hand back. It may sometimes produce a slight unintentional outwards hand twisting, owed to the gravity center position, which is too high to the hand. Thus, such a distribution makes the exoskeleton hard to be worn by the patient for a long time to have continuous assistance in ADLs. Instead, it could be more easily used for shorter rehabilitation exercises.

Finally, the three layers of the actuation box case improve the device usability and safety, enabling the wearer to change the exhausted batteries straightforwardly.

Table 1 reports some of the most significant state-of-the-art devices. It is worth noting that only a few of them are fully wearable, i.e., without any components dislocated from the hand, as Bouteraa et al. (2019) and Yurkewich et al. (2020) and *HandyRehab*. Unlike (Bouteraa et al., 2019), the new HES design eliminates cables and exploits a wireless bridge for sEMG signals transmission, as in Yurkewich et al. (2020) and *HandyRehab*. Compared to the commercially available *HandyRehab* the proposed HES is specifically tailored to the patient hand, and its finger-handling mechanisms result more streamlined. Finally, it is worth comparing the new prototype with *My-HERO* presented in Yurkewich et al. (2020). They have been designed for similar purposes but with different methods. Indeed, the proposed HES is based on rigid structures, while *My-HERO* is based on a soft one. The choice of using soft elements certainly reduces the weight and encumbrance of the system: *My-HERO* results, in fact, lighter and slimmer than the proposed exoskeleton. On the other side though, exploiting mechanical components increases the overall weight but generally ensure also greater kinematic accuracy and force effectiveness. Besides, the proposed design ensures that the palm and the lower surface of the fingers are mostly component-free; feature that, unlike the glove exploited in *My-HERO*, preserves the sense of touch. Finally, while *My-HERO* needs to establish and maintain a connection with a PC, the proposed system classifies the user intention internally, hence boosting the system portability and the user freedom of movement.

Overall, the novelty of this study lies in proposing a design that, differently from other state-of-the-art solutions, collects most of the primary crucial characteristics of assistive and rehabilitative devices (i.e., wearability, portability, safety, comfort, compliance, customization, force, and cost effectiveness). Nonetheless, the authors are well aware of the wide room for improvements left, in particular, regarding component miniaturization, thumb actuation, and independent finger movement.

## 6. Conclusions and Future Perspectives

**Figure 8:**
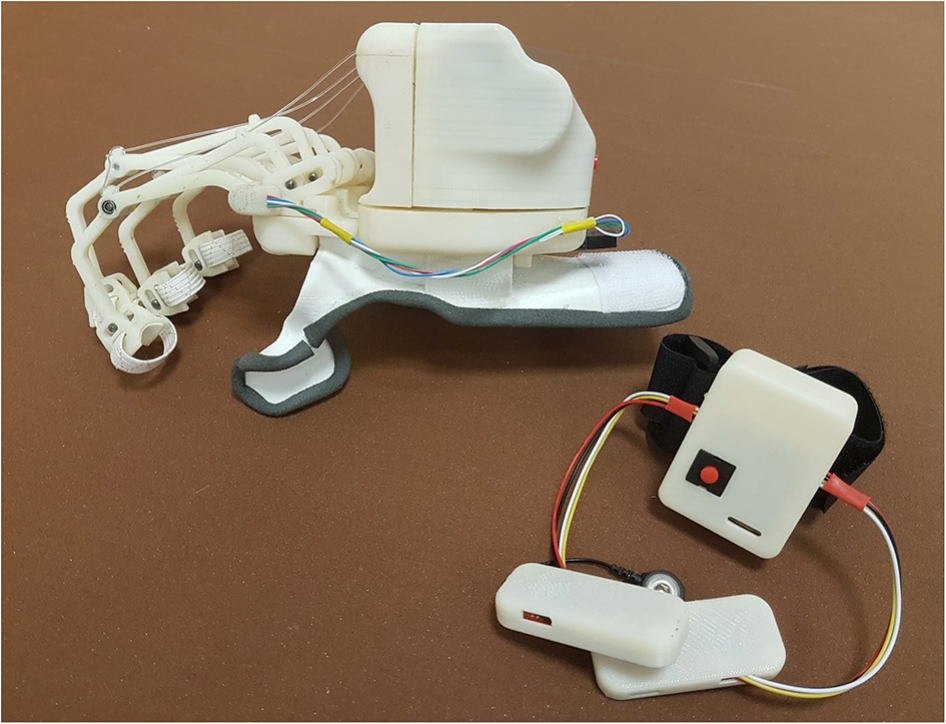
The figure shows the new architecture of the wearable HES developed at the MDM Lab.

The overall new HES mechatronic design, proposed in this article and shown in Figure 8, presents innovative and noteworthy aspects compared to the current state-of-the-art wearable robots for hand disabilities. The proposed solution has several strengths, of which some are inherited from the previous prototypes. The rigid mechanisms geometry and new splint-based interface are tailored to the patient hand and forearm to be as compliant as possible with its anatomy and natural movements, and thus comfortable for the patient. A single actuator exploitation considerably lightens the whole system, while using a toothed belt provides better stability to the motion transmission and acts as mechanical friction in emergency cases. Also, spaces inside the module on the hand back have been exploited to the best, which, along with not using any more cables for data transmission, prevent an annoying feeling of constraint on the forearm and increase safety. The sEMG-based intention recognition technique represents a highly intuitive way of managing the system motion. Besides, the new data transmission protocol allows for straightforward monitoring of the system status using any Bluetooth-compatible devices and thus also remotely rehabilitation treatments by performing tasks preset by the therapist. So, the new HES results to be customizable, compliant and comfortable for the patient. Its components placement does not add impairments to the user motions, also ensuring safety. The device is force effective, intuitive, and fully wearable and portable. Finally, the HES is affordable since it costs about 550ϵ, compact since it does not exceed a standard hand size, and lightweight since it weighs less than 550 g. Therefore, the primary requirements listed in section 1 have been met. The proposed enhancements allow to achieve a solution that results in helpful for the pilot study patient mainly in grasp movements, still having some difficulties in grip ones, depending on the item shape and sizes, especially in pinch movements. Also, it can be helpful in rehabilitation sessions. Concerning this point, it is worth noting that the possibility that the patient individually wears, uses, and removes the device depends on its conditions. If the patient cannot don the device alone, as in this study, the therapist presence is mandatory at home and in clinics.

Complete wearability and portability, safety, comfort, compliance, force effectiveness, customization, intuitiveness, and affordability are features encapsulated in this device, based on rigid mechanisms—unlike other soft solutions—and this represents the true novelty of the study.

Nevertheless, some flaws have still been and will be under investigation in the short-term future. Firstly, the cable-driven actuation needs to be replaced since, even if it can adapt well to the hand's complex kinematics, it allows active actuation only when opening. Such a solution is enough for the pilot study patient, but in enlarging the subjects that can use it, active actuation also during closing may be required. In addition, it sometimes presents a problematic reversal of motion—also happened during the experimental tests—mainly when too much cable has been unwound, thus requiring specific maintenance work to restart the HES. Second, the lack of a thumb-handling mechanism still limits usability since its opposition is crucial to achieving good dexterity in object handling. Intending to produce a complete device, the development of a thumb mechanism is now under investigation. Third, bearing in mind the mentioned considerations, the fingers' independent motion might be challenging to implement but crucial to allow different hand gestures, such as precision manipulation or pinching, and further improve ergonomics. Finally, the space on the hand back has been optimized, but the weight distribution worsens, especially for assistance purposes. Besides, the finger mechanism dimensions still prevent the HES use in confined spaces, e.g., turning a handle or pinching small items, as also happened during the ARAT.

Both component miniaturization and masses redistribution will undoubtedly improve the device usability, ergonomics, and comfort for all these reasons. Also, unlocking the wrist articulation might be considered by changing the HES support base and hand connection system. All these enhancements represent the core of the present and future research activities of the group.

## Data Availability Statement

The raw data supporting the conclusions of this article will be made available by the authors, without undue reservation.

## Ethics Statement

Ethical review and approval was not required for the study on human participants in accordance with the local legislation and institutional requirements. The patients/participants provided their written informed consent to participate in this study.

## Author Contributions

NS: conceptualization, writing, supervision, and experimentation. CB and FG: writing, supervision, and experimentation. MP, FV, YV, and AR: conceptualization and supervision. FB: conceptualization. MB: conceptualization and experimentation. All authors contributed to the article and approved the submitted version.

## Funding

This work has been supported by Don Carlo Gnocchi Foundation (Italy) and three research projects: the “HOLD” project funded by the University of Florence, the “HERMES” project funded by Fondazione CR Firenze, and the “BMIFOCUS” project funded by the Tuscany Region (POR FESR 2014–2020).

## Conflict of Interest

The authors declare that the research was conducted in the absence of any commercial or financial relationships that could be construed as a potential conflict of interest.

## Publisher's Note

All claims expressed in this article are solely those of the authors and do not necessarily represent those of their affiliated organizations, or those of the publisher, the editors and the reviewers. Any product that may be evaluated in this article, or claim that may be made by its manufacturer, is not guaranteed or endorsed by the publisher.

## Footnotes

^1^https://www.who.int/health-topics/disability (accessed August 31, 2021).

^2^https://www.who.int/news-room/fact-sheets/detail/noncommunicable-diseases (accessed August 31, 2021).

^3^https://handyrehab.com (accessed August 31, 2021).

^4^https://www.hkk-bionics.de/en/exomotion-en (accessed August 31, 2021).

^5^https://www.bioservo.com/healthcare/carbonhand (accessed August 31, 2021).

^6^https://www.neofect.com/us/neomano (accessed August 31, 2021).

^7^https://www.gloreha.com/?lang=it (Accessed August 31, 2021).

^8^https://www.bioniklabs.com/products/inmotion-arm (accessed August 31, 2021).
